# Genotypes of the Enterovirus Causing Hand Foot and Mouth Disease in Shanghai, China, 2012-2013

**DOI:** 10.1371/journal.pone.0138514

**Published:** 2015-09-23

**Authors:** Menghua Xu, Liyun Su, Lingfeng Cao, Huaqing Zhong, Niuniu Dong, Zuoquan Dong, Jin Xu

**Affiliations:** Department of Clinical Laboratory, Children’s Hospital of Fudan University, Shanghai, 201102, China; Institut Pasteur of Shanghai,Chinese Academy of Sciences, CHINA

## Abstract

Sporadic HFMD (hand foot and mouth disease, HFMD) cases and outbreaks caused by etiologic agents other than EV71 and CA16 have increased globally. We conducted this study to investigate the prevalence and genetic characteristics of enteroviruses, especially the non-EV71 and non-CA16 enteroviruses, causing HFMD in Shanghai. Clinical specimens were collected from patients with a diagnosis of HFMD. A partial length of VP1 was amplified with RT-PCR and subjected to direct sequencing. Phylogenetic analyses were performed using MEGA 5.0. The ages of the HFMD cases ranged from 3 to 96 months, and the male/female ratio was 1.41. The median hospital stay was 2.96 days. Up to 18.0% of patients had neurologic system complications such as encephalitis, meningoencephalitis or meningitis. Of the 480 samples, 417 were positive for enterovirus (86.9%) with RT-PCR. A total of 13 enterovirus genotypes were identified. The most frequent genotypes were CA6 (31.9%), EV71 (30.6%), CA16 (8.8%) and CA10 (7.5%). Infections with CA6, EV71, CA16 and CA10 were prevalent throughout the years of study, while the proportion of CA6 notably increased from Sep. 2012 to Dec. 2013. Phylogenetic analyses showed that EV71 strains belonged to the C4a subgenogroup and CA16 was identified as B1b subgenogroup. The CA6 strains were assigned to genogroup F, whereas the CA10 strains were assigned to genogroup D. Patients infected with CA6 were typically younger, had a shorter hospital stay and had a lower incidence of neurologic system complications when compared to patients infected with EV71. Our study demonstrates that the enterovirus genotypes causing HFMD were diversified, and there was an increasing prevalence of the non-EV71 and non-CA16 enteroviruses from 2012 to 2013. CA6 was the most predominant pathogen causing HFMD from Sep. 2012 to Dec. 2013, and it often caused relatively mild HFMD symptoms. Most severe HFMD cases were associated with EV71 infection.

## Introduction

Hand foot and mouth disease (HFMD), characterized by fever, and vesicular exanthema on the hands, feet, oral mucosa and buttocks, is a global and common disease in children, particularly in those less than 5 years old. Although HFMD is classically a mild disease and most infections resolve spontaneously, severe HFMD with fatal cardiopulmonary and neurologic complications have increasingly occurred in the Asian-Pacific region since 1997 and have attracted attention from many countries [1,2].

HFMD is caused by enteroviruses belonging to the genus of the Picornavirus family. Typically, enterovirus 71 (EV71) and coxsackievirus A16 (CA16) of the enterovirus A species have been the most common causative agents, and EV71 has become the dominant pathogen causing severe HFMD [1,3–8]. From 2008 to 2012, HFMD outbreaks associated with coxsackievirus A6 (CA6) and coxsackievirus A10 (CA10) have been increasingly reported in Finland, Singapore, Taiwan, Japan, the United States, and China, which has highlighted the importance of the non-EV71 and non-CA16 enteroviruses and the necessity of surveillance of the entire pathogen composition spectrum [9–15].

In China, large outbreaks of HFMD have occurred every year in different provinces in the past few years, and EV71 and CA16 are still the most frequent pathogens in co-circulation [5–8]. From 2012, the detection ratio of non-EV71 and non-CA16 enteroviruses in HFMD cases has indicated an upward trend [8,12,14–15]. In this study, we aimed to investigate the prevalence and genetic characteristics of these enteroviruses, especially the non-EV71 and non-CA16 enteroviruses causing HFMD, as well as their clinical characteristics, in Shanghai, China.

## Materials and Methods

### Sample collection

From 1 Jan 2012 to 31 Dec 2013, a total of 480 stool specimens were collected from patients hospitalized with HFMD who were diagnosed according to the Ministry of Health’s diagnostic criteria (http://www.nhfpc.gov.cn/yzygj/s3593g/201306/6d935c0f43cd4a1fb46f8f71acf8e245.shtml) at Children’s Hospital of Fudan University, Shanghai, China. The complications involving neurologic, respiratory or circulatory system diseases as a result of HFMD were defined in “A Guide to Clinical Management and Public Health Response for Hand Foot and Mouth Disease (HFMD)” (http://www.wpro.who.int/publications/docs/GuidancefortheclinicalmanagementofHFMD.pdf).

The study was approved by the Institutional Review Board of Children’s Hospital of Fudan University. All information and patient identifiers were kept anonymous to protect patient confidentiality. The information collected included the demographic data, any recorded symptoms or clinical information, clinical diagnoses, and laboratory findings. Since this study was a retrospective analysis from the patients who only received regular medical examination, and all the data obtained were de-identified, written consents from the patients were waived.

### Detection and genotyping of enteroviruses

Stool samples were collected and screened for enteroviruses. RNA were extracted from the supernatants of 10% (V/V) stool specimens by TRIzol (Invitrogen, CA, USA) and dissolved in 20 μl DEPC (Diethypyrocarbonate) water. The cDNA sample was synthesized by using PrimeScript TM RT kit (Takara, Dalian, China) in a total volume with 4 μl of RNA, 100 μmol random primers, and 2.5 U reverse transcriptase.

The detection and genotyping were performed by nested-PCR using the VP1 junction region of the enterovirus, as described [16]. For the first round, 2 μl of cDNA was used as a template with 0.5 μmol each of outer primers, 50 μmol of dNTP, and 0.75 U of ExTaq DNA polymerase in the following cycling conditions: 35 cycles of 94°C for 30 s, 54°C for 45 s, 72°C for 30 s, and a final incubation of 72°C for 7 min. A volume of 2 μl of the product was used for the second round of PCR with 0.5 μmol each of inner primers in a volume of 25 μl under the same PCR conditions described above. The PCR product was harvested for agarose gel electrophoresis, the DNA fragments were purified, and the nucleotide sequence of each PCR product was bi-directionally sequenced on a 3730 sequencer (Pekin-Elmer Applied Biosystems, Foster City, CA).

### Sequence analysis

The genetic identity of each PCR product was first determined by comparison with the standard strains in GenBank (US National Center for Biotechnology Information, NCBI). All the sequences obtained in this study were then deposited to GenBank with the given accession numbers KP717564-KP717665 for CA6, KP717666-KP717683 for CA10, KP717684-KP717686 for CA16 and KP717687-KP717759 for EV71. Multiple sequence alignment was conducted using ClustalW. Phylogenetic trees based on partial VP1 genes were built using the neighbor-joining method in the Molecular Evolutionary Genetics Analysis program, version 5.0 (MEGA 5.0). Gaps were treated as a complete deletion. Statistical support for each clade was assessed using bootstrap analysis with 1000 replicates. Genetic distances were calculated with the P-distance model. All the reference strains used were retrieved from Genbank database, which shared the highest identity with our sequences.

### Statistical analysis

For categorical variables, *x* ^*2*^ tests and/or Fisher’s exact tests were used, and Student’s *t* tests were used for continuous variables. *P* values were two-tailed, and they were considered significant if *P*≤0.05. Data analysis was performed with SPSS, version 18.0.

## Results

### Demographics of the patients

From Jan. 2012 to Dec. 2013, a total of 480 HFMD patients were recruited in the study with ages ranging from 3 months to 96 months (29.12±17.41 M). The vast majority, 94.2% of the patients, were less than 5 years of age, and the male/female ratio was 1.41. The median hospital stay was 2.96 days. Up to 18.0% of these patients had neurologic system complications such as encephalitis, meningitis and meningoencephalitis.

### Enterovirus genotypes

**Fig 1:**
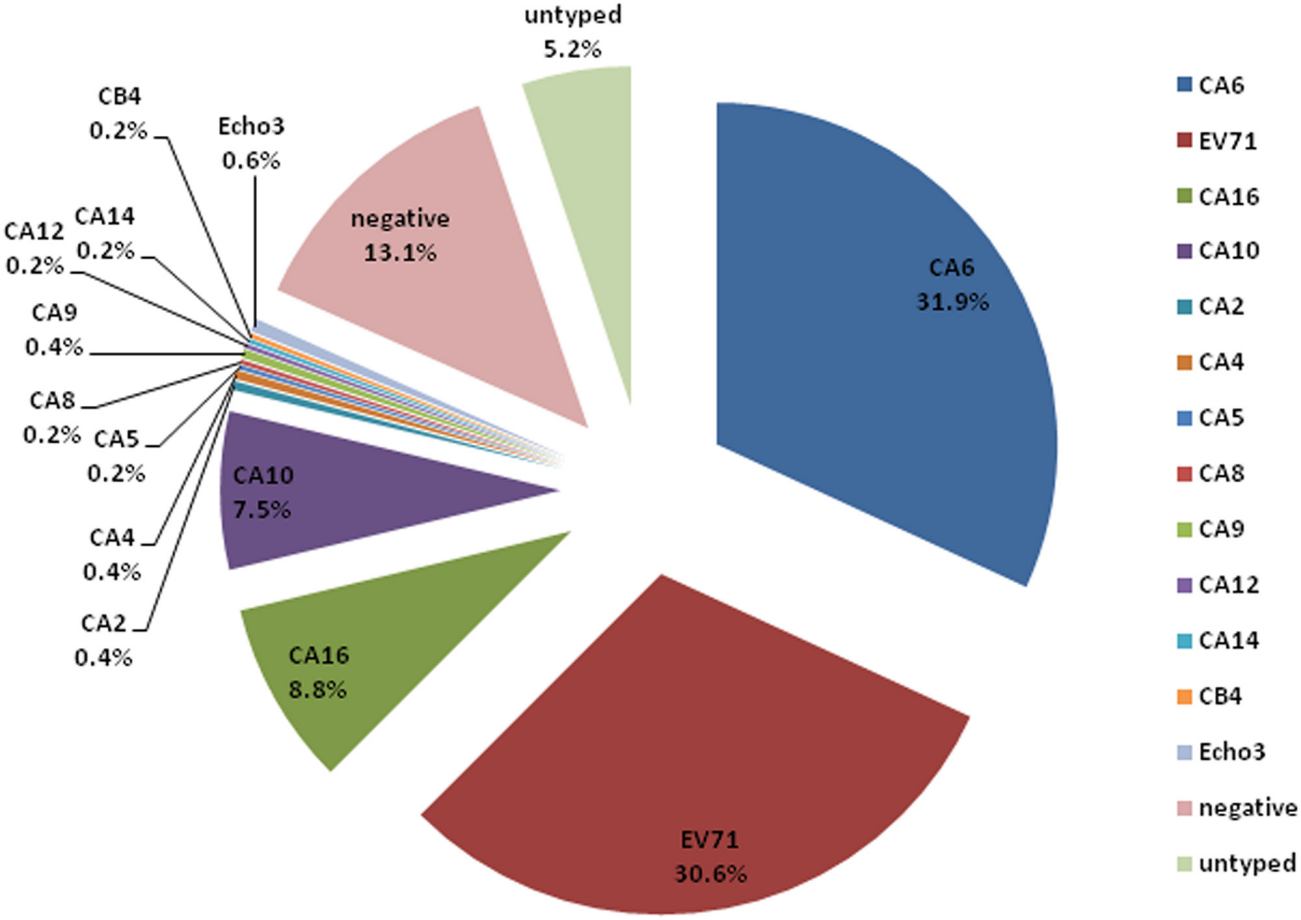
The genotypes of enteroviruses identified in HFMD patients from 2012 to 2013, Shanghai, China.

Among 480 samples, 417 (86.9%) were positive for enterovirus. A total of 13 genotypes were detected by sequencing. The most prevalent genotype was CA6 (31.9%, 153/480), followed by EV71 (30.6%, 147/480), CA16 (8.8%, 42/480), CA10 (7.5%, 36/480) and Echo 3 (0.6%, 3/480), CA2 (0.4%, 2/480), CA4 (0.4%, 2/480), CA9 (0.4%, 2/480), CA5 (0.2%, 1/480), CA8 (0.2%, 1/480), CA12 (0.2%, 1/480), CA14 (0.2%, 1/480), and CB4 (0.2%, 1/480) (Fig 1). In 2012, 8 genotypes were identified including EV71 (38.3%), CA6 (20.4%), CA16 (15.8%), CA10 (7.5%), Echo3 (0.8%) and CA4 (0.4%), CA8 (0.4%), CA9 (0.4%). In 2013, 12 genotypes were detected, including CA6 (43.3%), EV71 (22.9%), CA10 (7.5%), CA16 (1.7%), CA2 (0.8%), CA4 (0.4%), CA5 (0.4%), CA9 (0.4%), CA12 (0.4%), CA14 (0.4%), CB4 (0.4%), and Echo3 (0.4%).

**Fig 2:**
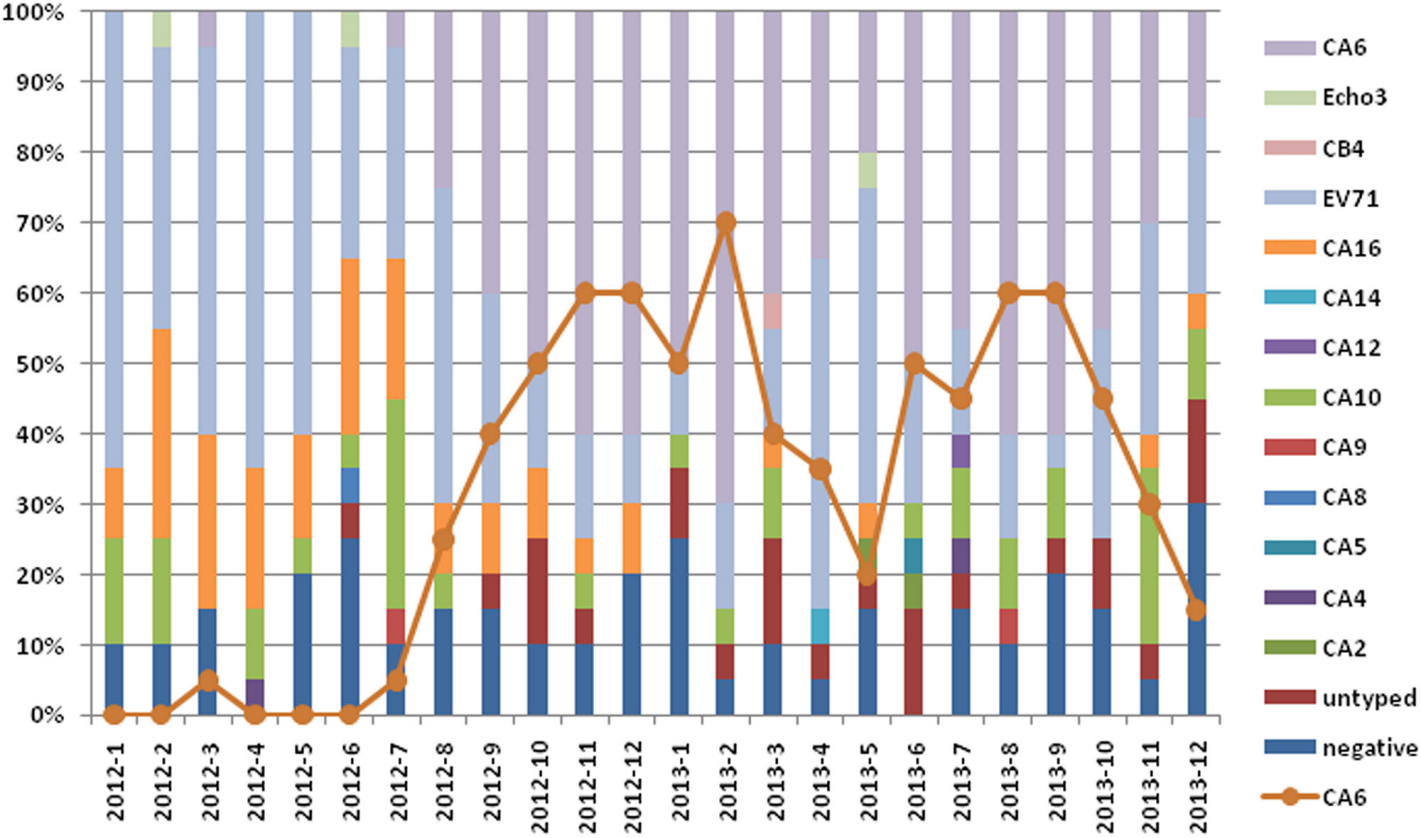
The temporal distribution of enterovirus genotypes identified in HFMD patients from 2012 to 2013, Shanghai, China.

Genotypes CA6, EV71, CA16 and CA10 were detected throughout the years of 2012 to 2013. The proportion of CA6 notably increased from Sep. 2012 to Dec. 2013, while the detection ratio of EV71 showed a decreasing trend (Fig 2).

### Phylogenetic analysis of enterovirus genotypes

**Fig 3:**
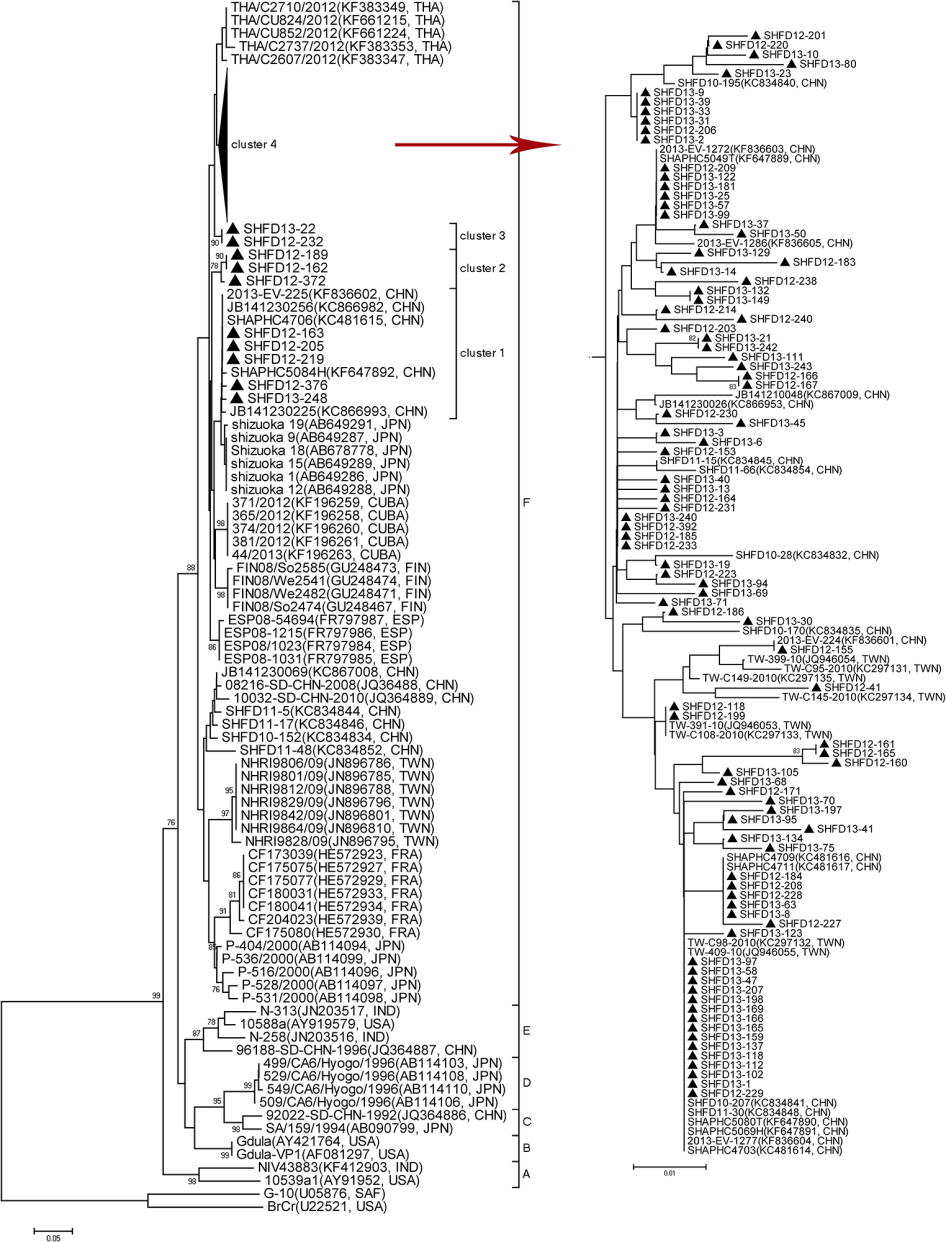
Phylogenetic analysis with the neighbor-joining method with a 1000-bootstrap re-sampling based on the alignment of the 368 nucleotide VP1 gene of 102 Shanghai CA6 strains. The strains labeled with ▲ were obtained in our study.

Nucleotide sequence alignment of the partial VP1 gene was performed using 102 Shanghai CA6 strains isolated from the HFMD patients in this study. The partial VP1 genes of CA6 strains in our study showed 92.7%-99.7% similarity, and the pairwise distances among them ranged from 0.000 to 0.073. According to the criteria established for genogrouping EV71 and CA16 by calculating genetic distance, the CA6 strains could be classified into A-F genogroups by the selected genetic distance threshold as 0.09 (Fig 3) [17,18]. Genogroups A-E mainly included some earlier CA6 strains from all over the world. Genogroups A, B, and E comprised a few strains from India and the USA and one strain from Japan, while C and D comprised a few strains from Japan and one strain from China in 1992. Genogroup F comprised some strains from foreign areas and strains circulating in China from 2008 to 2013.

All of the CA6 strains identified in our study were categorized into genogroup F, which could be further divided into four clusters. The majority of our CA6 strains displayed a close genetic relationship with the Chinese strains identified from other provinces in China and Taiwan, during 2010 and 2013, and these strains were also similar to strains from Thailand in 2012, Finland and Spain in 2008, Japan in 2011 and Cuba in 2012, which were associated with onychomadesis subsequent to HFMD.

**Fig 4:**
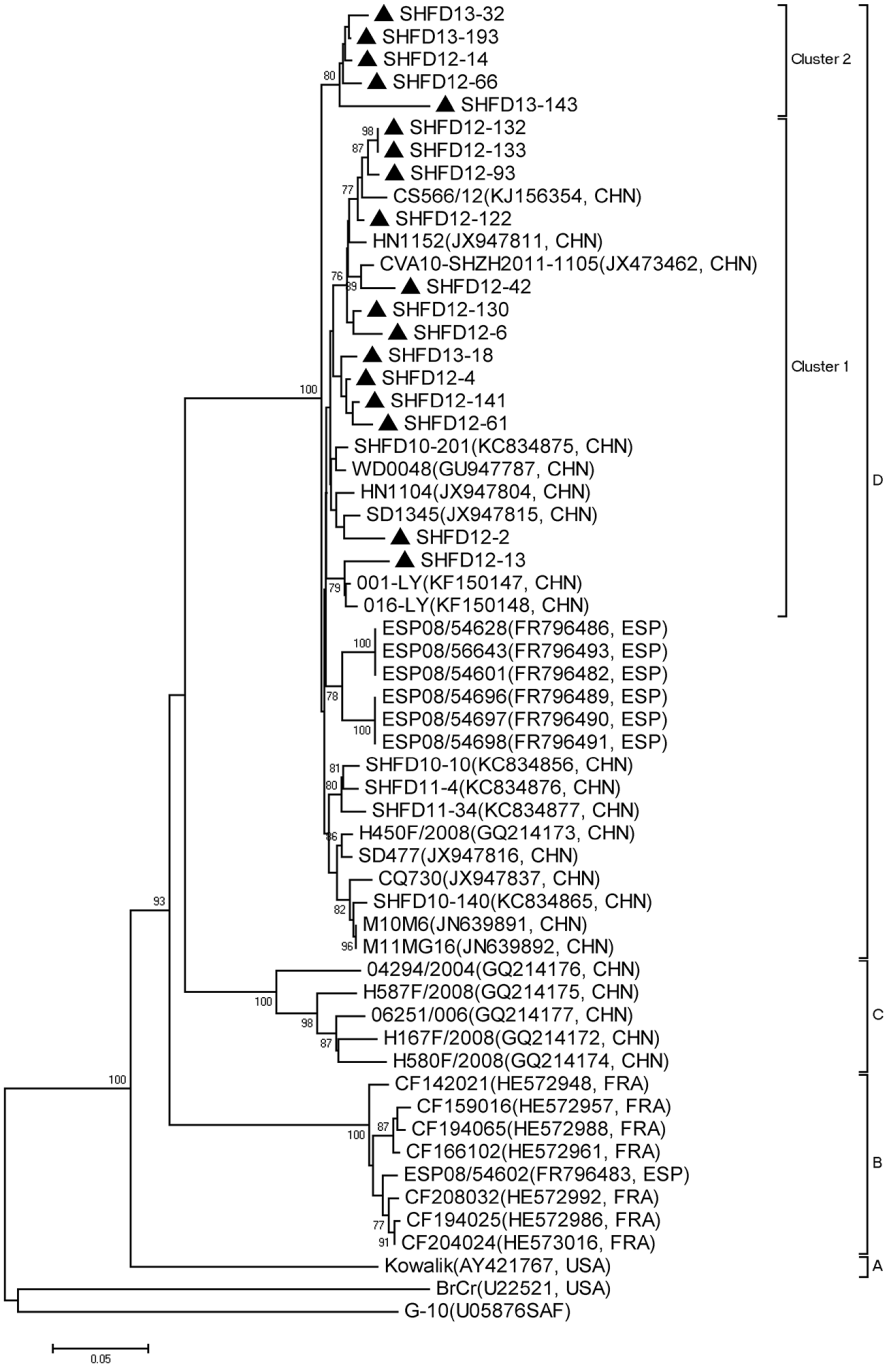
Phylogenetic analysis with the neighbor-joining method with a 1000-bootstrap re-sampling based on the alignment of the 394 nucleotide VP1 gene of 18 Shanghai CA10 strains. The strains labeled with ▲ were obtained in our study.

The CA10 strains in our study showed 90.8%-99.1% similarity, and the pairwise distance among them ranged from 0.001 to 0.091. According to the criteria established for genogrouping EV71 and CA16 by calculating genetic distance, the CA10 strains could be assigned to 4 genogroups (A-D) by the selected genetic distance threshold as 0.09 (Fig 4) [17,18]. Phylogenetic tree showed that all 18 Shanghai CA10 strains belonged to genogroup D. All Chinese CA10 strains were classified into genogroup C and D. Genogroup C included earlier isolated CA10 strains, while genogroup D included the strains in this study and the other most recent CA10 strains from other provinces in China from 2008 to 2012, together with Spanish strains from 2008. The CA10 strains in this study in cluster 1 had a close genetic relationship with other CA10 strains isolated from other provinces in China, while another five formed a cluster 2 independently.

**Fig 5:**
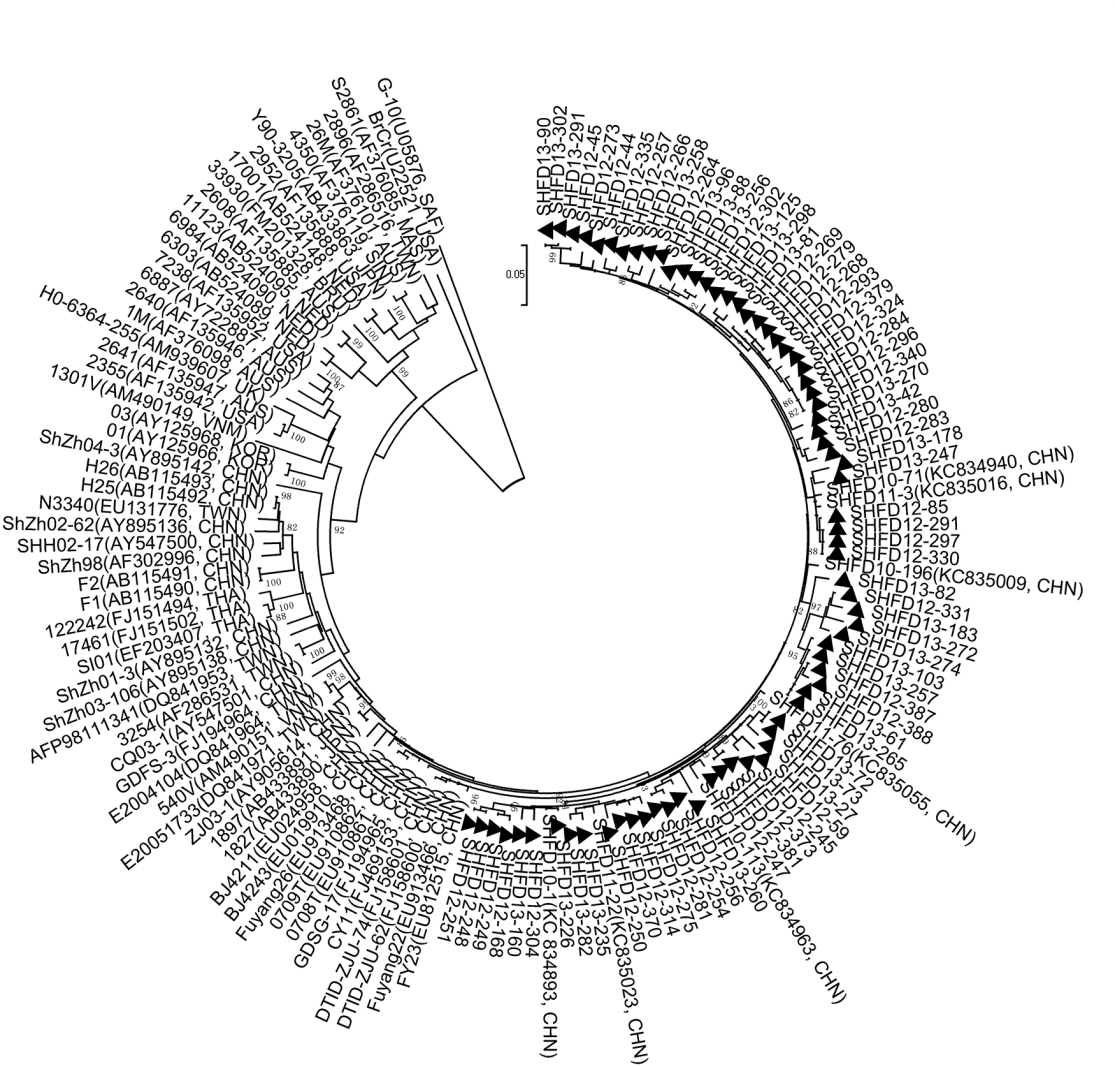
Phylogenetic analysis with the neighbor-joining method with a 1000-bootstrap re-sampling based on the alignment of the 468 nucleotide VP1 gene of 73 Shanghai EV71 strains. The strains labeled with ▲ were obtained in our study.

**Fig 6:**
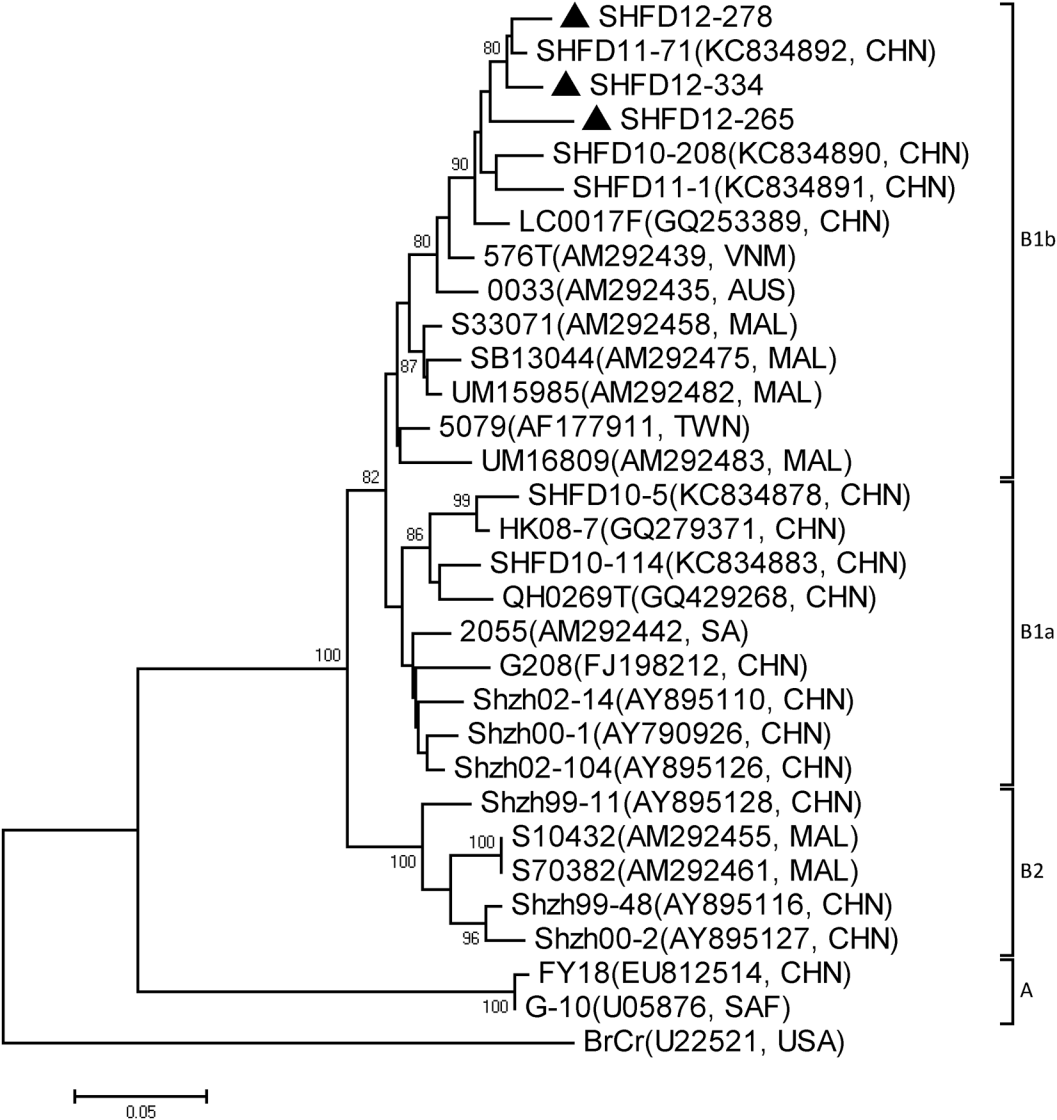
Phylogenetic analysis with the neighbor-joining method with a 1000-bootstrap re-sampling based on the alignment of the 399 nucleotide VP1 gene of 3 Shanghai CA16 strains. The strains labeled with ▲ were obtained in our study.

Both phylogenetic trees were also constructed with EV71 and CA16. The results revealed that the two viruses circulated continuously in 2012 and 2013. All the EV71 strains identified in this study belonged to subgenogroup C4a, with a high similarity of 93.9%-99.8% between the sequences (Fig 5). Phylogenetic analysis indicated that the partial CA16 VP1 sequences in this study were grouped to B1b with 91.6%-96.8% similarity (Fig 6). Together, C4a of EV71 and B1b of CA16 are the predominant genogroups circulating in Shanghai in the period of the study.

### Clinical features

After genotyping, we divided patients into 4 groups infected with different enterovirus genotypes and collected their clinical information. Patients with EV71 infection had a significantly older age, longer mean hospital stay, and higher incidence of neurologic complications than the patients infected with CA6, CA10 or CA16 (*P*<0.05) (Table 1). EV71-infected patients had a much higher incidence of fever, startle, limb shaking and neck rigidity than other groups. The EV71 infection group was more likely to have abnormal numbers of white blood cells in the cerebrospinal fluid (CSF), which was a significant difference from the other groups (*P*<0.05). An elevated serum C-reactive protein (CRP) level >40 mg/L was noted in a substantial proportion of the patients in the CA6 and CA10 infection groups. The CA16 infection group had a higher incidence of abnormality of cardiac troponin I (cTnI) when compared to the EV71 infection group (*P*<0.05) (Table 2). Furthermore, the data based on a telephone interview showed that in addition to the typical presentations of skin eruptions on the hands, feet and mouth, some of the patients with CA6 infection had nail abnormalities such as onychomadesis.

**Table 1:** Demographics Features and diagnoses of HFMD patients.

Characterics	CA6 (n = 152)	CA10 (n = 35)	CA16 (n = 42)	EV71 (n = 144)	Total (n = 472)
**Sex, male/female**	1.64:1	1.77:1	1.8:1	1.67:1	1.41:1
**Age, mouths**	23.68±13.51^#^	22.63±11.93^#^	29.19±16.66^#^	36.04±20.11	29.12±17.41
**Hospital stay, days**	2.53±0.99^#^	2.57±0.78^#^	2.62±1.69^#^	3.51±1.69	2.96±1.55
**Complications, n(%)**					
** Respiratory system**	37 (24.3)^#^	5 (14.29)	10 (23.81)	20(13.89)	88 (18.64)
** Digestive system**	8 (5.26)	1(2.86)	1 (2.38)	2 (1.39)	15(3.18)
**Circulatory system**	4 (2.63)	0(0)	3 (7.14)	2 (1.39)	9(1.91)
** Neurologic system**	12 (7.89)^#^	0 (0)^#^	2 (4.76)^#^	62 (43.06)	85 (18.01)

^#^: Compared with EV71 infection group, *P* < 0.05.

**Table 2:** Comparison of the clinical symptoms and laboratory findings of HFMD patients with different enterovirus infection.

Characterics	CA6 (n = 152)	CA10 (n = 35)	CA16 (n = 42)	EV71 (n = 144)	Total (n = 472)
**Clinical symptoms**					
** Fever (%)**	137(90.13)	33(94.29)	36(85.71)^#^	137(95.14)	412(87.29)
** Oral ulcer (%)**	140(92.11)	32(91.43)	39(92.86)	129(89.58)	406(86.02)
** No rash (%)**	2(1.32)	0(0)	2(4.76)	2(1.39)	7(1.48)
** Cough (%)**	31(20.39)	5(14.29)	11(26.19)	20(13.89)	79(16.74)
** Vomiting (%)**	27(17.76)	10(28.57)	12(28.57)	38(26.39)	102(21.61)
** Headache (%)**	0(0)	0(0)	1(2.38)	2(1.39)	4(0.85)
** Startle (%)**	20(13.16)^#^	7(20.00)	9(21.43)	34(23.61)	80(16.95)
** Febrile seizure (%)**	6(3.95)	3(8.57)^#^	0(0)	1(0.69)	15(3.18)
** Limb shaking (%)**	3(1.97)^#^	1(2.86)^#^	3(7.14)	26(18.06)	36(7.63)
** Neck rigidity (%)**	0(0)^#^	0(0)	0(0)	5(3.47)	5(1.06)
**Laboratory findings**					
** WBC(×10** ^**9**^ **/L)**	9.69±4.15	9.44±3.69	10.12±4.01	9.33±2.92	9.54±3.67
** WBC>12×10** ^**9**^ **/L (%)**	38(25.00)	9(25.71)	11(26.19)	25(17.36)	95(20.13)
** ALT(U/L)**	12.55±16.35	9.97±4.40	11.00±6.05	12.40±24.84	13.52±32.49
** ALT>40 (%)**	3(1.97)	0(0)	0(0)	4(2.78)	11(2.33)
** AST(U/L)**	24.45±11.82	22.40±7.14	24.12±7.14	23.66±17.10	25.08±24.89
** AST>40 (%)**	6(3.95)	1(2.86)	2(4.76)	8(5.56)	23(4.87)
** CK-MB(U/L)**	44.66±26.00^#^	41.97±28.13	41.73±22.85	37.92±23.39	41.56±27.72
** CK-MB>40 (%)**	58(38.16)	8(22.86)	17(40.48)	41(28.47)	143(30.3)
** CRP>40 (%)**	22(14.47)^#^	11(31.43)^#^	1(2.38)	2(1.39)	45(9.53)
** CTnI abnormality (%)**	9(5.92)	2(5.71)	5(11.90)^#^	4(2.78)	26(5.51)
** CSF WBC>10×10** ^**6**^ **/L (%)**	1/16(6.25)^#^	0/8(0)^#^	0/8(0)^#^	52/63(82.54)	61/108(56.48)
** CSF Pro>500U/L (%)**	2/16(12.50)	0/8(0)	1/8(12.50)	11/63(17.46)	17/108(15.74)

^#^: Compared with EV71 infection group, *P* < 0.05.

## Discussion

HFMD, first reported in New Zealand in 1957 as a common infectious disease, has since become a serious threat to public health in China. Multiple, large-scale outbreaks have occurred in the past few decades and have caused numerous deaths every year (http://www.chinacdc.cn/tjsj/).

Since the late 1990s, HFMD outbreaks have often been reported in the Asian-Pacific region, in countries including Malaysia, Japan, Perth, Taiwan and China, in which EV71 and CA16 occupied the predominant position [8,19–22]. In China, surveillance of HFMD has mostly focused on EV71 and CA16. However, other enterovirus genotypes, especially CA6 and CA10, have now become the most common pathogens of HFMD, replacing EV71 and CA16 in some regions in recent years [9–15]. In this study, a total of 13 enterovirus genotypes were detected, and high circulation rates of CA6 and CA10 were found. The higher detection rate of CA6, when compared to that of EV71 and CA16, suggests it was the most common causative pathogen in the years of 2012 and 2013, while EV71 remains a major pathogen.

CA6- and CA10-related HFMD outbreaks occurred in some Asian and European countries recently. In 2008 in Singapore, the most prevalent pathogen was demonstrated to be CA6 and CA10, with a total detection rate of 35.3% [10]. CA10-related HFMD outbreak was also reported in 2008 in Spain [23]. In 2008, an outbreak of HFMD occurred in Finland with the causative pathogen found to be CA6 [9]. The prevalences of CA6 and CA10 were as high as 28% and 39.9%, respectively, in 2010 in France [24]. In 2012 in India, CA6 was the major pathogen of HFMD, with the rare detection of CA10 and EV71 [25]. CA6 also caused outbreaks of HFMD in Taiwan in 2010 and in Japan in 2011 as a primary pathogen [11,12].

In this study, of the 13 enterovirus genotypes detected from 2012 to 2013, CA6 (31.9%) replaced EV71 and CA16 as the most causative agent of HFMD in Shanghai, while the detection rate of CA10 was as high as 7.5%. To clarify the genetic characteristics, phylogenetic trees based on partial VP1 sequences from CA6 and CA10 strains were constructed. The representative sequences used in this study, that available from Genbank, still formed an independent cluster in the same genogroup as that in the other studies [26,27]. All our CA6 strains were quite divergent and showed four different clusters, which indicates there may have different transmission chains in Shanghai. The majority of CA6 strains belonged to cluster 4 and were much closer to strains isolated from Taiwan and other areas of China. Meanwhile, some CA6 strains formed their own cluster, with a high bootstrap value. Phylogenetic analysis of CA10 strains demonstrated that most CA10 strains in this study were close to strains isolated from other provinces in China, which mostly indicated that the prevalent CA10 strains in this study were genetically homogeneous from other strains in China, while 5 CA10 strains also formed their own cluster, with a high bootstrap value of 78%. The independent CA6 and CA10 strains, forming their own clusters, indicated they may evolve to acquire their own characteristics. For further study, a robust spatiotemporal phylogenetic analysis of CA6 and CA10, based on complete VP1 sequences, will be needed.

The genogroup of EV71 in Shanghai during 2012 and 2013 was C4a, which is most closely related to the strains isolated in different provinces in China. In China, except for one C3 genogroup strain isolated in 1997 and five A genogroup strains isolated in 2008, all the EV71 strains have been classified into the C4a genogroup in outbreaks after 2004 [28–31]. CA16 has been divided into genogroups A, B1a, B1b and B2 [17]. In this study, the CA16 strains were grouped into B1b, together with CA16 strains obtained in our previous report, revealed co-circulation of B1a and B1b from 2010 to 2013 in Shanghai, with no significant variation from the strains isolated in other areas of China [7,31–33]. This study further confirmed the predominant position of the EV71 C4a genogroup and the CA16 B1 genogroup in China [29–34].

Most HFMD infections are self-limited and do not require hospitalization. Previous studies have demonstrated that EV71 more frequently causes severe complications in young children than other enteroviruses and often leads to acute flaccid paralysis and brainstem encephalitis associated with poor outcomes [35–37]. In this study, patients infected with EV71 were the oldest in age, had the longest hospital stays and the highest incidences of severe complications.

Recent studies in Finland, Taiwan, France, American and China have shown that patients infected with CA6 express clinical symptoms such as onychomadesis and extensive skin ulcerations that are different from those infected with other enterovirus genotypes [9, 11,13–14,24,38]. Onychomadesis is a non-inflammatory, painless nail change that is idiopathic or results from a wide range of systemic diseases or drug exposures. The mechanism of onychomadesis remains unclear, and to some extent, this may indicate the enhancement of the virulence of CA6. In this study, we found that HFMD patients infected with CA6 had a younger age of onset, a shorter hospital stay, and a lower incidence of neurologic complications when compared to those patients infected with EV71. In addition, we also observed obvious onychomadesis in the CA6-caused HFMD patients, as appeared in Finland, Taiwan, Japan and other provinces in China, with the loss of nails occurring 1–2 months after initial symptoms [9,11,12,14]. Further study should be intensified to isolate CA6 strains and observe their features of pathogenicity and regionalism.

In summary, this study strongly indicated that CA6 was the predominant HFMD pathogen from late 2012 to 2013, which emphasized the necessity of surveillance of all the enterovirus genotypes causing HFMD in China. While the HFMD vaccine was developed for EV71, these changes in pathogenic compositions of an HFMD outbreak should affect future vaccine development and clinical management.
